# Interaction between *Borrelia miyamotoi* variable major proteins Vlp15/16 and Vlp18 with plasminogen and complement

**DOI:** 10.1038/s41598-021-84533-x

**Published:** 2021-03-02

**Authors:** Frederik L. Schmidt, Valerie Sürth, Tim K. Berg, Yi-Pin Lin, Joppe W. Hovius, Peter Kraiczy

**Affiliations:** 1 Institute of Medical Microbiology and Infection Control, University Hospital of Frankfurt, Goethe University, Frankfurt, Germany; 2 Division of Infectious Diseases, Wadsworth Center, New York State Department of Health, Albany, NY USA; 3 Department of Biomedical Science, State University of New York at Albany, Albany, NY USA; 4 Center for Experimental and Molecular Medicine, Academic Medical Center, Amsterdam Infection and Immunity Institute, Amsterdam, The Netherlands

**Keywords:** Immunology, Microbiology, Diseases, Pathogenesis

## Abstract

*Borrelia miyamotoi*, a relapsing fever spirochete transmitted by Ixodid ticks causes *B. miyamotoi* disease (BMD). To evade the human host´s immune response, relapsing fever borreliae, including *B. miyamotoi*, produce distinct variable major proteins. Here, we investigated Vsp1, Vlp15/16, and Vlp18 all of which are currently being evaluated as antigens for the serodiagnosis of BMD. Comparative analyses identified Vlp15/16 but not Vsp1 and Vlp18 as a plasminogen-interacting protein of *B. miyamotoi*. Furthermore, Vlp15/16 bound plasminogen in a dose-dependent fashion with high affinity. Binding of plasminogen to Vlp15/16 was significantly inhibited by the lysine analog tranexamic acid suggesting that the protein–protein interaction is mediated by lysine residues. By contrast, ionic strength did not have an effect on binding of plasminogen to Vlp15/16. Of relevance, plasminogen bound to the borrelial protein cleaved the chromogenic substrate S-2251 upon conversion by urokinase-type plasminogen activator (uPa), demonstrating it retained its physiological activity. Interestingly, further analyses revealed a complement inhibitory activity of Vlp15/16 and Vlp18 on the alternative pathway by a Factor H-independent mechanism. More importantly, both borrelial proteins protect serum sensitive *Borrelia garinii* cells from complement-mediated lysis suggesting multiple roles of these two variable major proteins in immune evasion of *B. miyamotoi*.

## Introduction

*Borrelia *(*B.*)* miyamotoi* is a vector-borne human pathogenic spirochete transmitted by ixodid ticks and causes the so-called hard tick-borne relapsing fever (HTBRF) or *B. miyamotoi* disease (BMD)^1–3^. Initially isolated from *Ixodes persulcatus* in Hokkaido^4^, *B. miyamotoi* has also been detected in other *Ixodes (I.)* species such as *I. ricinus, I. persulcatus, I. scapularis,* and *I. pacificus*^4–8^. These ticks also carry other tick-borne pathogens, including, but not limited to Lyme Disease (LD) spirochetes, *Anaplasma phagocytophilum*, and *Babesia microti*, raising a concern of multiple tick-borne pathogens that can be transmitted next to or simultaneously with *B. miyamotoi*^9^. Upon infection, *B. miyamotoi* may hematogenously spread to distant tissues and causes acute high-grade fever accompanied with headache, fatigue, myalgia, arthralgia, chills, and nausea^10–13^. Compared to relapsing fever caused by *B. duttonii*, the soft ticks-transmitted relapsing fever, *B. miyamotoi* infection only occasionally triggers numerous episodes of recurrent high fever^10,14–18^. In addition, a detailed comparative sequence analyses revealed that *B. miyamotoi* identified in ticks collected from an individual region exhibit a very low sequence variability. In contrast, sequences obtained from ticks collected from different geographical regions with distinct *Ixodes* tick species as vectors showed considerable diversity, allowing the classification of *B. miyamotoi* into at least three separate clades^8,19–23^.

Multiple episodes of recurrent high fever, the hallmark presentation of relapsing fever, are thought to be caused by the humoral immune responses to spirochete surface antigens^24^, the decrease and resurgence of *Borrelia* populations, including *B. miyamotoi*, producing antigenically variant surface proteins^11,25–27^. This system consists of polymorphic genes encoding for immunodominant variable major proteins (Vmps), which are dispersed as silent promoter-less *vmp* gene cassettes on archival linear plasmids^25,26^. By a nonreciprocal gene transfer of a silent *vmp* cassette into the single *vmp* expression site, antigenically diverse Vmps are sequentially produced by a single bacterial cell. Based on the molecular mass, Vmps are devided into two families: the variable small proteins (Vsp) of ~ 20 kDa and variable large proteins (Vlp) of ~ 40 kDa whereas at least 19 *vsp* and *vlp* sequences have been identified in *B. miyamotoi* LB-2001 of which six encode for a full signal peptide, and 38 *vlp* as well as 10 *vsp* genes have been identified in strain Izh-4^23,25^. A recent study investigating the role of Vmp’s in *B. miyamotoi* pathogenesis demonstrated that anti-Vsp1 IgG antibodies were able to efficiently eliminated Vsp1 producing spirochetes by a complement-dependent mechanism from the bloodstream of C3H/HeN mice^27^. Moreover, this study also revealed that patients elicited robust antibody responses to several Vmps including Vsp1, Vlp15/16, and Vlp18 suggesting that the humoral immune response, even transiently, can induce clearance of the infection. Interestingly, Vmps of *B. hermsii* bind to extracellular-matrix glycosaminoglycans (GAGs) such as heparin and chondroitin sulfate^28,29^, and thus appear to be associated with spirochete adhesion.

In addition to humoral immune responses, other host systems are exploited by RF borreliae to promote dissemination^30,31^. One of these systems is the fibrinolytic pathway, which requires the presence of plasminogen. Previously, it was suspected that recruitment of host-derived plasminogen is a potential strategy for *Borrelia* invasion and may facilitate spirochete´s dissemination and migration into extravascular tissues by utilizing the proteolytic activity of plasmin^32–34^. Plasminogen is synthesized in the liver as an inactive proenzyme and circulates in the bloodstream (~ 2.4 µM) and many extravascular fluids. This protein consists of a N-terminal pre-activation peptide, five lysine-binding, disulfide-bonded kringle domains (K1-K5) and a C-terminal serine protease domain^35^. Proteolytic cleavage of plasminogen by urokinase-type plasminogen activator (uPA) or endogenous tissue-type plasminogen activator (tPA) results in the conversion of this protein to its active version, plasmin^36^. Owing to its broad substrate specificity, plasmin also degrades many components of the extracellular matrix, matrix metalloproteases and complement components^37^.

Another host system that RF borreliae modulate is complement, the first line of host immune defence^38,39^. Like a well-organized network consisting of membrane-bound and fluid-phase molecules, this system is tightly controlled by diverse regulators and inhibitors to avoid destruction of host cells. The complement cascade can be activated by three distinct routes, the alternative (AP), the classical (CP), and the lectin pathway (LP). Independent of the route of activation, highly reactive C3b molecules affixed to the microbial surface are generated by the so-called C3 convertases to flag and prepare invading microorganisms for opsonophagocytosis. Upon progression, binding of C3b to the C3 convertases of the AP or CP/LP result in the formation of the C5 convertases that cleave C5 into C5b and C5a. C5b attached to the foreign surface initiates the terminal sequence in which C6, C7, C8, and several molecules of C9 are assembled together to form the C5b-9 complex or membrane attack complex (MAC). Concerning regulation of the AP, Factor H (FH) plays a crucial role to prevent excessive and an uncontrolled complement activation on self surfaces^40,41^. By serving as cofactor for Factor I (FI), FH either inactivates surface-bound C3b or accelerate the dissociation of the C3 convertase of the AP from the foreign surface (decay-accelerating activity) and thereby completely terminate all down streaming activation processes. As a cofactor for FH and the C4b binding protein, FI controls all three pathways due to its proteolytic cleavage activity to C3b and C4b.

A sophisticated strategy developed by RF borreliae, including *B. miyamotoi*, to escape complement and fibrinolytic pathway is to produce several outer surface proteins that bind to complement inhibitors or plasminogen^42–47^. Such binding down-regulates complement-mediated killing and activates plasminogen-mediated ECM component degradation, which is thought to confer efficient dissemination. In light of the severe manifestations caused by *B. miyamotoi* in some patients and the tropism to neural tissues, we aimed at investigating the molecular mechanism utilized by *B. miyamotoi* to traverse endothelial barriers to reach deeper tissues by interacting with host-derived plasminogen. In this study, we aimed to functionally characterize Vsp1, Vlp15/16, and Vlp18 of *B. miyamotoi* concerning their potential to bind to host plasminogen and to display complement-inhibitory activities.

## Results

### Identification of potential plasminogen-binding proteins of *B. miyamotoi* HT31

CbiA has previously been identified as a multifunctional outer surface protein of *B. miyamotoi* HT31 exhibiting complement and plasminogen binding properties and thereby promoting resistance to complement-mediated killing and enabling spirochetes to degrade extracellular matrix proteins^43,46^. In the present study, we set out to examine if there are additional plasminogen-binding proteins of *B. miyamotoi* by selecting three variable major proteins including Vsp1, Vlp15/16, and Vlp18 previously evaluated as promising candidates for the serodiagnosis of BMD^27,48^ due to their structural relatedness to OspC of *B. burgdorferi* known to bind plasminogen^49^.

**Figure 1:**
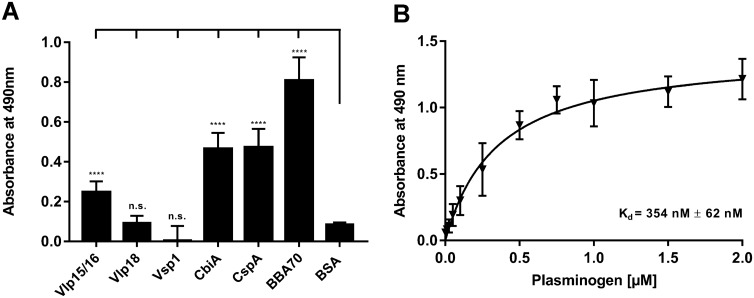
Identification of plasminogen-binding proteins in *B. miyamotoi*. (**A**) Binding of plasminogen to recombinant proteins by ELISA. Vlp15/16, Vlp18, Vsp1, CbiA, CspA, BBA70, and BSA (5 µg/ml each) immobilized were incubated with 10 µg/ml plasminogen. Bound plasminogen was detected using a polyclonal antibody. (**B**) Dose-dependent binding of plasminogen to Vlp15/16. Vlp15/16 (5 µg/ml) were immobilized and incubated with increasing concentrations of plasminogen. Binding curve and dissociation constant were approximated via non-linear regression, using a one-site, specific binding model using GraphPad Prism version 7. Data represent means and standard deviation of at least three different experiments, each conducted in triplicate. ****p ≤ 0.0001, n.s., no statistical significance, one-way ANOVA with post-hoc Bonferroni multiple comparison test (confidence interval = 95%).

Initially, binding of plasminogen to Vsp1, Vlp15/16, and Vlp18 was assessed by ELISA. In addition, three proteins previously identified as plasminogen-interacting molecules were used as positive controls namely CbiA of *B. miyamotoi* HT31 as well as CspA and BBA70 of *B. burgdorferi* LW2^46,50,51^ while bovine serum albumin (BSA) served as negative control. To detect binding of plasminogen, purified His_6_-tagged borrelial proteins were immobilized on microtiter plates (5 μg/ml each) and the protein–protein interaction was then assessed by using a polyclonal anti-plasminogen antibody. As shown in Fig. 1A, Vlp15/16 as well as the borrelial control proteins CbiA, CspA, and BBA70 significantly bound plasminogen by ELISA. In contrastVsp1, Vlp18, and BSA did not bind to the host protein. Additional analysis disclosed a dose-dependent binding of Vlp15/16 to plasminogen whereby the interactions revealed a strong affinity in the nanomolar range with a calculated dissociation constant of *K*_*d*_ = 354 nM (± 62 nM) (Fig. 1B).

### Influence of ionic strength on the binding of plasminogen to Vlp15/16

**Figure 2:**
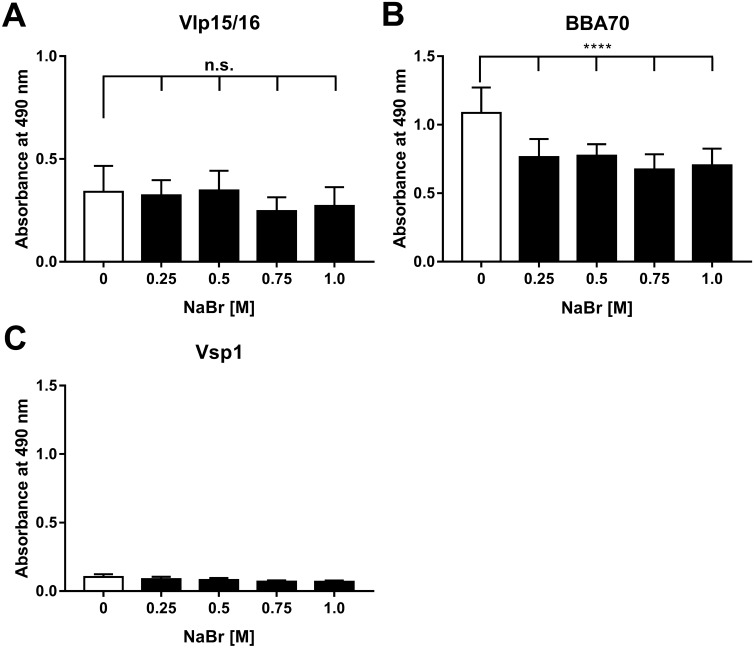
Influence of plasminogen binding by ionic strength. To determine influence of ionic strength on the plasminogen binding, Vlp15/16, BBA70 (positive control), and Vsp1 (negative control) (5 µg/ml each) were immobilized and incubated with plasminogen in the presence of increasing concentrations of NaBr. Bound plasminogen was detected using a polyclonal antibody (1:1000). Data represent means and standard deviation of at least three independent experiments, each conducted in triplicate. ****p ≤ 0.0001, one-way ANOVA with post-hoc Bonferroni multiple comparison test (confidence interval = 95%). n.s., no statistical significance.

Having demonstrated binding of Vlp15/16 to plasminogen, we sought to characterize the nature of the protein–protein interaction in more depth. To this end, an ELISA with NaBr was conducted known to adversely affect electro-static interactions between interacting proteins^52^. Vlp15/16, Vsp1 (negative control), and BBA70 (positive control) were immobilized and subsequently incubated with a mixture containing plasminogen and increasing concentrations of NaBr. Of note, NaBr was used instead of NaCl because chloride anions are known to alter the conformation of plasminogen^52^ and thus, may influence the accessibility of plasminogen to a potential ligand. Binding of plasminogen to Vlp15/16 remained completely unaffected even at the highest concentration applied (1 M) (Fig. 2). As expected, increasing concentrations of NaBr affected the BBA70-plasminogen interaction^51^ but did not influenced the interaction of Vsp1 with plasminogen. These findings suggested that electrostatic forces do not play a role in the nature of the interaction of Vlp15/16 to plasminogen.

### Roles of lysine residue for the binding of plasminogen to Vlp15/16

**Figure 3:**
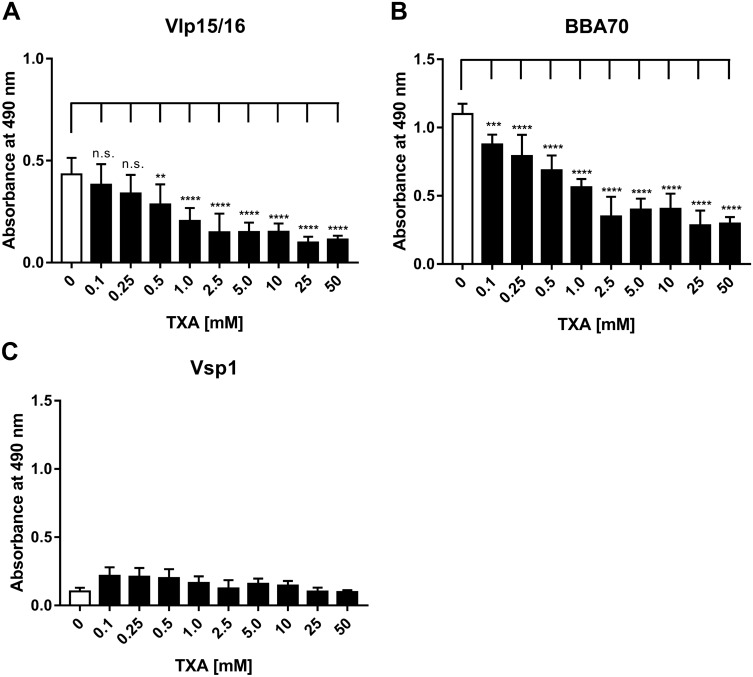
Involvement of lysine residues in the interaction of Vlp15/16 with plasminogen. To determine involvement of lysine residues increasing concentrations of the lysine analog tranexamic acid (TXA), Vlp15/16, BBA70, and Vsp1 (5 µg/ml each) were immobilized and incubated with plasminogen in the presence of increasing concentrations of tranexamic acid. Bound plasminogen was detected by a polyclonal antibody (1:1000). Three independent experiments were conducted in triplicate and graphs represent means ± SEM. *p ≤ 0.033; **p ≤ 0.002; ***p ≤ 0.0002; ****p ≤ 0.0001; n.s., no statistical significance, one-way ANOVA with post-hoc Bonferroni multiple comparison test (confidence interval = 95%).

To further assess the involvement of lysine residues in the interaction of plasminogen with Vlp15/16, additional binding assays were performed by using tranexamic acid as a lysine analog^53^. Lysine-binding sites located within the five kringle domains, in particular kringle domain 1, are mainly involved in the interaction of plasminogen with components of the ECM, host proteins and receptors as well as diverse bacterial proteins^54–56^. Vlp15/16, Vsp1, and BBA70 were incubated with plasminogen dissolved in increasing concentrations of tranexamic acid and binding was detected using a specific anti-plasminogen antibody. Concerning the Vlp15/16-plasminongen interaction, increasing concentrations of tranexamic acid significantly reduced the binding of the host protein in a dose-dependent manner, suggesting that lysine residues mediate binding of plasminogen to Vlp15/16 (Fig. 3A). As previously shown, binding of BBA70 to plasminogen was readily affected in the presence of merely 0.1 mM tranexamic acid (Fig. 3B)^51^. As expected, the absorbance values did not change when Vsp1 as an additional protein was employed (Fig. 3C).

### Plasminogen bound to Vlp15/16 is converted to plasmin

**Figure 4:**
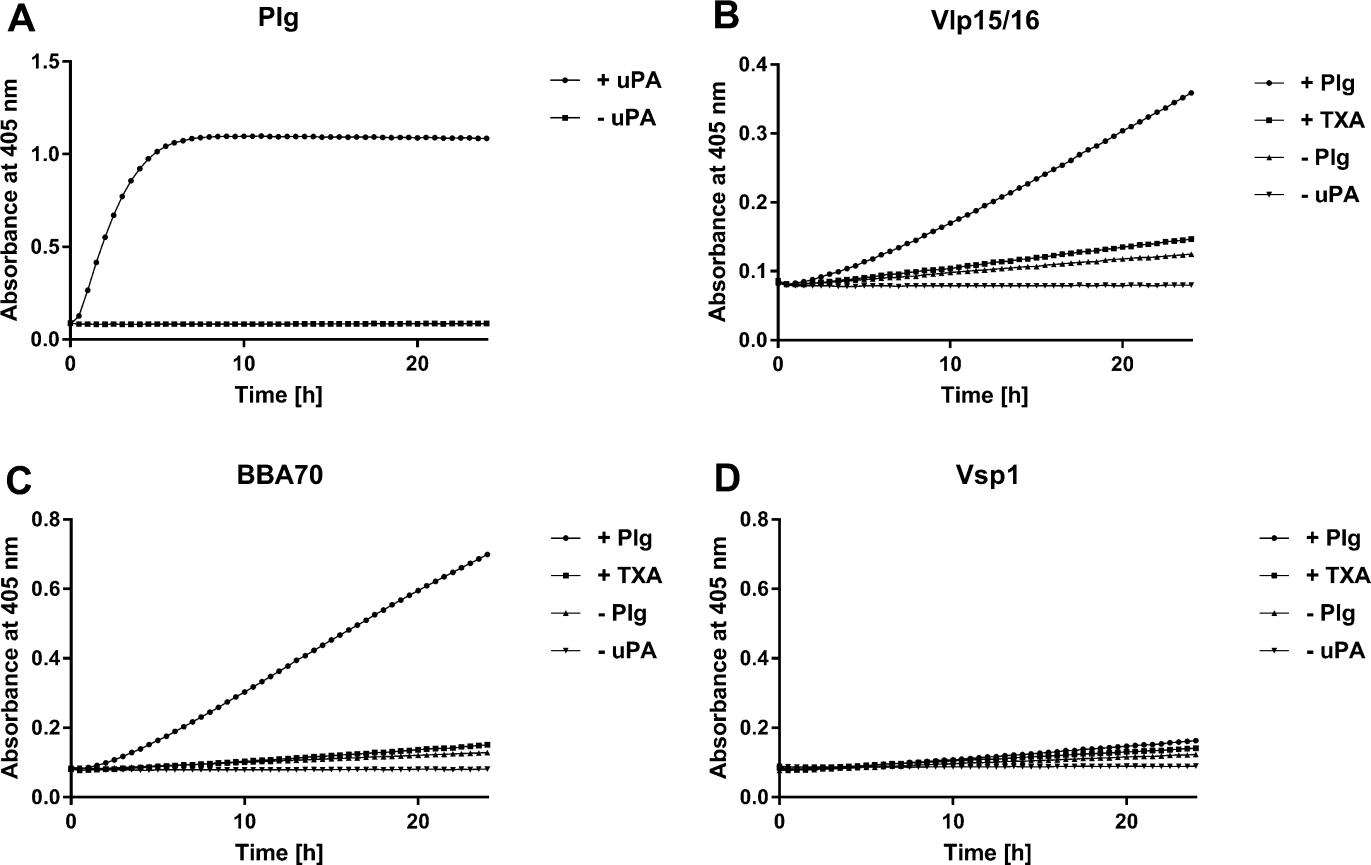
Plasminogen is converted to plasmin by uPA when bound to Vlp15/16. Microtiter plates were either coated with 5 µg/ml of plasminogen (Plg) (**A**) or with 5 µg/ml of purified Vlp15/16 **(B)**, BBA70 (**C**) or Vsp1 (**D**). The plasminogen-coated wells were then incubated with (filled circle) or without uPA (filled square). The immobilized borrelial proteins were subsequently incubated with 10 µg/ml plasminogen. Following incubation uPA and the chromogenic substrate D-Val-Leu-Lys-p-nitroanilide dihydrochloride (S-2251) was added (filled circle). Control reactions included 50 mM of the lysine analog tranexamic acid (T) (filled square) or omitted plasminogen (filled triangle) or uPA (filled inverted triangle), respectively. Microtiter plates were incubated at RT for 24 h and absorbance at 405 nm was measured at 30 min intervals. At least three independent experiments were conducted, each in triplicate. Data shown are from a representative experiment.

Plasminogen, an active precursor of the serine protease plasmin, circulate in human blood as and is activated by tissue-type (tPA) or urokinase-type plasminogen activator (uPA)^36^. Independent from the activator, the cleavage site of plasminogen bound to a ligand (e.g. fibrinogen or a protein of bacterial origin) needs to be accessible to maintain its proteolytic function as serine protease. To demonstrate whether plasminogen bound to Vlp15/16 and BBA70 is converted to plasmin, proteins were first immobilized to microtiter plates. As negative control proteins, Vsp1 and BSA were also included. Following incubation with plasminogen, uPA was added to the reactions together with the plasmin-specific chromogenic substrate D-Val-Leu-Lys-p-nitroanilide dihydrochloride (S-2251) and cleavage of the molecule was monitored for a period of 24 h. As demonstrated in Fig. 4A, reactions with purified plasminogen revealed a strong proteolytic activity to S-2251 after conversion of plasminogen to plasmin in the presence of uPA, while no cleavage occurred when uPA was omitted from the reaction mixture. Furthermore, degradation of S-2251 was also detected when plasminogen was bound to Vlp15/16 indicating that bound plasminogen was converted to plasmin (Fig. 4B). By employing BBA70, known to exhibit a strong affinity to plasminogen, a clear signal was monitored after 24 h of incubation (Fig. 4C). In contrast, additional control reactions containing Vsp1 did not result in degradation of the chromogenic substrate (Fig. 4D). As expected, no cleavage of the chromogenic substrate was observed in the presence of tranexamic acid (preventing binding to plasminogen) or when plasminogen or uPA were omitted from the reactions. These findings indicate that plasminogen is readily accessible to uPA and converted to active plasmin upon binding to Vlp15/16 and BBA70.

### Vlp15/16 and Vlp18 of *B. miyamotoi* inhibit activation of the alternative pathway by a FH-independent mechanism

**Figure 5:**
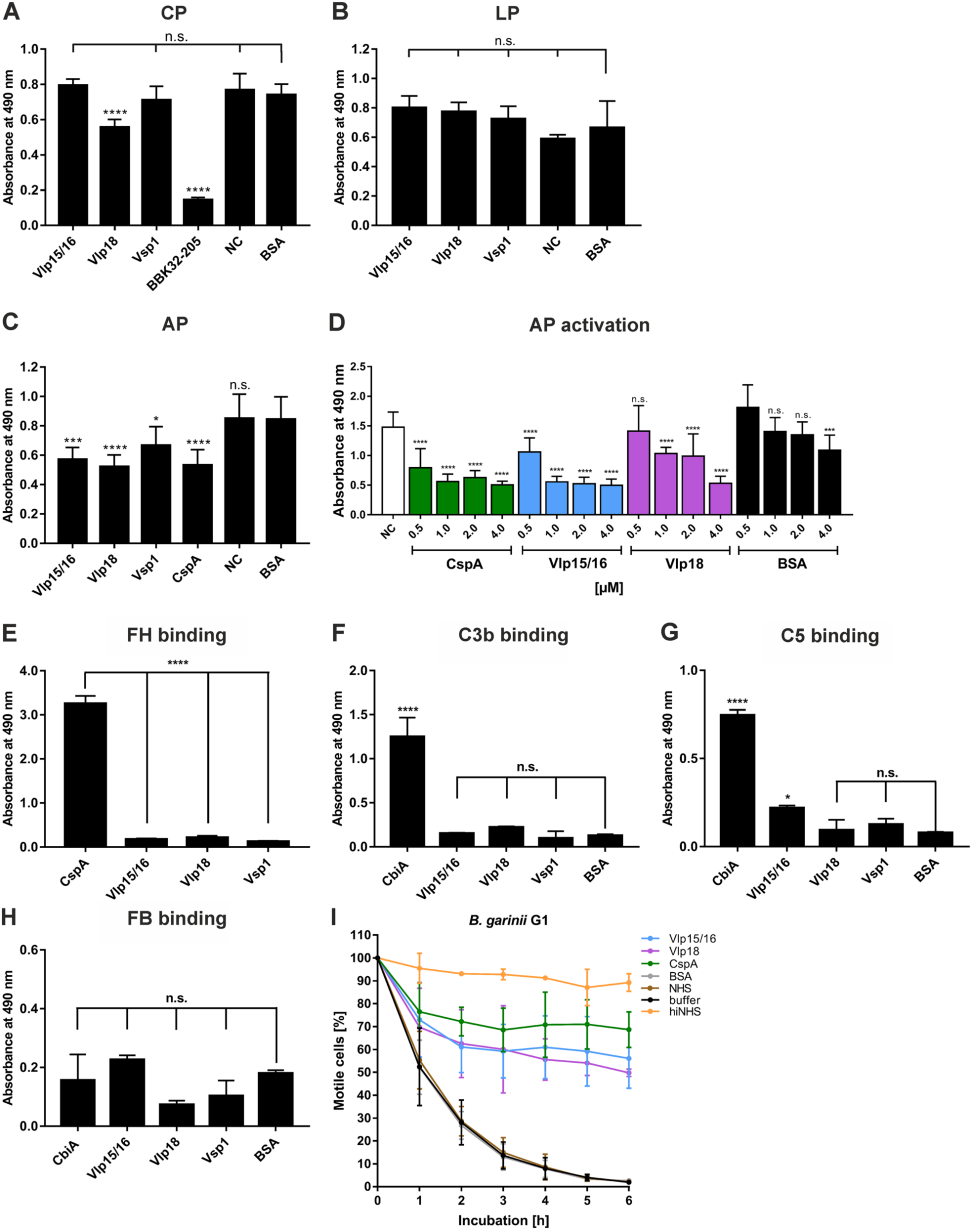
Vlp15/16 and Vlp18 inhibit complement activation independently from binding to FH and protect serum sensitive *B. garinii* from complement-mediated lysis. Assessment of the inhibitory capacity of Vmp’s on complement activation by an ELISA-based assay. Microtiter plates immobilized with IgM (CP) (**A**), mannan (LP) (**B**), and LPS (AP) (**C**) were incubated with NHS pre-incubated with the purified proteins or BSA (10 µg each). Formation of the MAC was detected by a monoclonal anti-C5b-9 antibody. Dose-dependent inactivation of the AP (**D**). Microtiter plates immobilized with LPS and after blocking, wells were incubated with NHS pre-incubated with increasing concentrations of the borrelial proteins or BSA. Formation of the MAC was detected by a monoclonal anti-C5b-9 antibody. Binding of recombinant borrelial proteins to FH (**E**), C3b (**F**), C5 (**G**), and Factor B (FB) **(H)**. Microtiter plates were coated with recombinant proteins, incubated with purified FH, C3b, and FB, respectively, and antigen–antibody complexes were detected using specific antisera. All experiments were performed at least three times, with each individual test carried out in triplicate. *p ≤ 0.033; ***p ≤ .0002; ****p ≤ 0.0001, one-way ANOVA with Bonferroni post-hoc test (confidence interval = 95%). n.s., no statistical significance; NC, negative control. Protection of serum sensitive *B. garinii* by Vlp15/16 and Vlp18 (**I**). NHS pre-incubated with 4 µM of the respective proteins was added to 1 × 10^8^ spirochetes and viability and motility of borrelial cells were determined at 0, 1, 2, 3, 4, 5, and 6 h of incubation. At least three independent experiments were conducted and ± SEM were calculated.

Diverse bacterial plasminogen-binding proteins as well as plasmin(ogen) play a role in inactivating complement^33,47,57,58^. We thus aimed to examine the ability of all three variable major proteins (Vlp15/16, Vlp18, and Vsp1) in inhibiting the activation of complement cascades. Microtiter plates prepared with either IgM, mannan or LPS for initiating activation of the CP, LP, and AP, respectively, were incubated with the respective purified proteins previously treated with NHS. These proteins included Vlp15/16 as well as the C-terminal fragment of BBK32 (BBK32-205) and CspA from *B. burgdorferi* as the control proteins for the CP and AP, respectively (Fig. 5). Following incubation with the NHS-treated reactions, the generation of the MAC was measured by applying a neoepitope-specific antibody as described previously^59^. As shown in Fig. 5A, among the borrelial proteins investigated, only Vlp18 inhibited the CP to some extent while none of the three *B. miyamotoi* proteins affected activation of the LP (Fig. 5B). Concerning the AP, Vlp15/16 and Vlp18 significantly inhibited activation of this pathway similar to CspA in a dose-dependent manner (Fig. 5C,D). However, Vsp1 appeared to slightly affected activation of the AP. BSA used as an additional control did not influence complement activation to at least 2 µM. These findings indicate that Vmp´s are able to interact with complement in different ways by influencing activation of the CP to some extent (Vlp18) or strongly affected the AP (Vlp15/16 and Vlp18).

Having demonstrated AP inactivation capacity of Vlp15/16 and Vlp18, we next assessed binding of FH, C3b, and Factor B (FB) to these *B. miyamotoi* proteins. CspA was used as a control protein known to be the key FH-binding protein of *B. burgdorferi*^60^ and as a further control, CbiA was utilized known to bind C3b^43^. Microtiter plates were coated with the purified proteins and binding of FH, C3b, C5, and FB was detected by specific polyclonal antibodies. Except CspA, none of the proteins analysed bound to the key regulator of the AP (Fig. 5E) as well as C3b, C5, and FB (Fig. 5F,G,H) indicating that Vlp15/16 and Vlp18 neither terminate AP activation by a FH dependent mechanism nor by direct binding to the central component C3b. To assess the functional role of Vlp15/16 and Vlp18 for serum resistance of *B. miyamotoi*, a serum bactericidal assay with serum sensitive *B. garinii* cells as surrogate were conducted. As depicted in Fig. 5I, both proteins protected spirochetes from bacteriolysis to up to 45% compared to cells treated with 50% NHS. Complement inhibition was observed when CspA was assayed under the same conditions and incubation with heat-inactivated NHS did not influence viability of the spirochetes over the whole incubation period. Complement was still active when NHS was pre-incubated with 4 µM of BSA or with Tris/HCl as buffer control indicating that complement activation was attenuated by Vlp15/16 and Vlp18, respectively.

## Discussion

In this study, we identified Vlp15/16 as a novel ligand for the host-derived serine protease plasminogen. Our functional characterization revealed, that Vlp15/16 displayed a strong affinity for plasminogen and that the nature of the interaction is partially mediated by lysine residues but not influenced by ionic strength. Furthermore, plasminogen bound to Vlp15/16 was converted to active plasmin in the presence of uPA^61^. Moreover, our comparative analysis also discloses that Vlp15/16 and Vlp18 exhibit complement inhibitory capacity on the CP and AP. In summary, the plasminogen binding properties of Vlp15/16 as well as the complement inhibitory activity of Vlp15/16 and Vlp18 associated with their immune evasion properties may enable *B. miyamotoi* to overcome innate as well as adaptive immunity and may promote spirochetes dissemination.

A common strategy of invading pathogens to combat and evade host responses is to camouflage themselves with potent proteolytic enzymes, regulators, and inhibitors continuously circulating in the blood often as inactive precursors or zymogens^30,33,62^. Moreover, the recruitment of host-derived proteases such as plasminogen endows pathogens lacking such powerful enzymes with a broad-spectrum proteolytic activity (e.g. *Borrelia* species) and may increase the invasiveness of those bacteria that are categorically equipped with diverse proteases (e.g. *Candida albicans*, *Staphylococcus aureus*, *Streptococcus pyogenes*)^63–65^. Generally, plasminogen-binding proteins can be grouped into two main categories: first, proteins which are anchored in the outer membrane and exposed to the external environment and second, molecules that reside in the cytoplasm or periplasm^62^. Here, we identified an additional plasminogen-interacting protein of *B. miyamotoi*, Vlp15/16, which can be grouped as a typical category 1 outer surface protein exhibiting crucial function in immune evasion (Fig. 1). Independent of their localization (cellular or membrane-associated) bacterial proteins characterized so far display, which a strong affinity to plasminogen, e.g. CbiA (347 nM) of *B. miyamotoi*, enolase (23 nM) and BBA70 (125 nM) of *B. burgdorferi*; CipA (36 nM) and Ef-Tu (57 nM) of *Acinetobacter baumannii*; enolase (360 nM) of *Mycobacterium tuberculosis*^46,51,66–69^.

In nature, the interaction of plasmin is mediated via the lysine-binding sites within the kringle domains K1, K2, K4, and K5, lysine and/or arginine residues in the respective ligand, e.g. fibrin^70–72^ or diverse bacterial proteins^46,51,67,68,73–77^. Especially, C-terminal lysine residues or lysine-rich motifs have been identified as essential determinants for the plasminogen-protein interaction as previously demonstrated for enolase of *S. pneumoniae*, CipA of *A. baumannii*, ErpP and BBA70 of *B. burgdorferi*^51,67,75,76^. The Vlp15/16 protein contains a single lysine residue at the C-terminus and several clusters of lysines all of which are randomly distributed over the whole molecule as also shown among Vlp15/16 orthologs of *B. miyamotoi* (Supplementary Figs. 1 and 2). However, substitution of the C-terminal lysine residues by alanine using in vitro mutagenesis was unsuccessful, thus we did not further narrow down the structural determinants responsible for the Vlp15/16-plasminogen interaction. The lysine analog tranexamic acid was initially used to identify the high affinity binding sites in the five kringle domains of plasminogen^70^. As mentioned, our analyses support the notion that lysine residues are more relevant for the Vlp15/16-plasminogen interaction as previously shown for BBA70 (Fig. 3). Such an impact of tranexamic acid on the interaction with plasminogen has previously been observed for CspA of *B. burgdorferi* as well as for HcpA of *B. recurrentis* and BhCRASP-1 of *B. hermsii*^42,45,77^. The noticeable effect of tranexamic acid revealed that the interaction of plasminogen with Vlp15/16 is preferentially mediated by lysine residues rather than other factors, e.g. electrostatic forces. As clearly shown, NaBr had a negligible effect on Vlp15/16 binding even at the highest concentration utilized (1.0 M) (Fig. 2). These findings indicate that binding of plasminogen to Vlp15/16 depends on hydrophobic interaction as also demonstrated for the Lpp protein of *Escherichia coli*, CbiA of *B. miyamotoi*, and BBA70 of *B. burgdorferi*^46,51,78^ or complement regulator C4BP^79^.

Accessibility of plasminogen to the plasminogen activators uPA or tPA is a prerequisite for the conversion to proteolytic active plasmin. Here we demonstrated that plasmin promotes degradation of the chromogenic substrate D-Val-Leu-Lys-*p*-nitroanilide dihydrochloride (S-2251) upon binding to Vlp15/16 in the presence of uPA (Fig. 4). Furthermore, cleavage of the chromogenic substrate was either completely or at nearly abrogated in control reactions in which uPA was omitted or when tranexamic acid was added suggesting that plasminogen bound to its ligands is fully accessible.

Pathogenic bacteria developed numerous artful strategies to escape from recognition and elimination by the human complement system^30,31,39^. Here we show that both, Vlp15/16 and Vlp18 inhibited the AP, and Vlp18 also affected the CP to some extent. Unexpectedly, activation of the AP was inhibited by both Vmp´s independently from the interaction with complement regulator FH known to be a common immune evasion mechanism utilized by diverse bacteria including LD borreliae and relapsing fever spirochetes (Fig. 5D,E)^11,80^. *S. aureus* has developed a sophisticated strategy to inhibited AP activation. At least four bacterial proteins from *S. aureus* including Efb, Ehp, Sbi, and SCIN have previously been described to interact with either the C3b thioester domain (TED) [Efb, Ecb (also known as Ehp), and Sbi] or the C3 convertases of the AP and CP (SCIN) and thereby efficiently counteract the bactericidal effects of complement^81–84^. Our findings suggest that Vlp15/16 and Vlp18 might strike similar targets to combat AP activation like the staphylococcal protein SCIN^81^ as both molecules did not interact with C3b, C5 or FB (Fig. 5F,G,H). Additional analyses revealed that plasminogen bound to Vlp15/16 did not recruit C3b, C3, C5 or FB as a potential mechanism to target complement (Supplementary Fig. 3). Thus, further studies are required to disclose whether Vmp´s (Vlp15/16) of *B. miyamotoi* as well as other RF borreliae inhibit the complement cascade at the level of C3 activation and amplification by affecting the formation of the C3 convertase. The data presented herein also revealed that purified Vlp15/16 and Vlp18 are able to protect serum sensitive *B. garinii* cells from complement-mediated lysis by up to 45% (Fig. 5I) indicating that both proteins participate in serum resistance and play a role in immune evasion of *B. miyamotoi*. Furthermore, a recent study showed that a *vmp*-deficient relapsing fever borreliae triggered bacteraemia in SCID mice at levels 28-fold lower than the parental wild-type spirochete^85^. This result raises a possibility that Vmp contributes to spirochete survival in the host bloodstream by evading a response independent of adaptive immunity, which is, in fact, supported by our finding of complement evasion and plasminogen binding conferred by this protein.

In summary, we here describe additional roles of variable major proteins Vlp15/16 and Vlp18 involved in immune evasion. Overall, elucidating the complex interplay of these multifactorial proteins in vivo will certainly proof to be an important step towards understanding the pathogenic processes triggered by the relapsing fever spirochete *B. miyamotoi*.

## Material and methods

### Bacterial strains and culture conditions

*B. miyamotoi* HT31 was cultivated at 33 ºC in a modified Kelly-Pettenkofer medium supplemented with 40% fetal calf serum as previously described^86^. *B. garinii* G1 was cultured until mid-exponential phase (5 × 10^7^ cells per ml) at 33 °C in Barbour-Stoenner-Kelly (BSK-H) medium (Bio&SELL, Feucht, Germany). For production of hexahistidine (His_6_)-tagged proteins, *Escherichia (E.) coli* BL21 (DE3) cells (New England Biolabs, Frankfurt, Germany) grown in yeast tryptone (YT) broth at 37 °C were utilized.

### Human serum, proteins, and antibodies

Human serum (NHS) was collected from healthy blood donors as described previously^50^. Human glu-plasminogen was purchased from Haematologic Technologies (Essex Junction, VT, USA) and urokinase plasminogen activator (uPA) (Merck, Darmstadt, Germany) were used for the activation of plasminogen to plasmin. The chromogenic substrate S-2251 (D-Val-Leu-Lys *p*-nitroanilide dihydrochloride) were from Sigma-Aldrich (Steinheim, Germany). Factor H, Factor B, C3b, and C5 were purchased from Complement Technology (Tyler, TX, USA). Polyclonal anti-plasminogen antibody was purchased from Acris Antibodies (Herford, Germany), and the monoclonal anti-plasminogen antibody (clone 10-V-1) was from Calbiochem, Merck, Darmstadt, Germany). The polyclonal anti-FH and anti-C3 antibody were obtained from Merck Biosciences (Bad Soden, Germany) and the polyclonal anti-C5, anti-Factor B antibody as well as the neoepitope-specific monoclonal anti-C5b-9 antibody was from Quidel (San Diego, CA, USA). The mouse anti-His antiserum was obtained from Novagen (Merck Darmstadt, Germany) and Qiagen (Hilden, Germany) and the horseradish peroxidase (HRP)-conjugated immunoglobulins were purchased from Dako (Hamburg, Germany).

### Generation of His_6_-tagged proteins

The generation of His_6_-tagged CspA and BBA70 from *B. burgdorferi* LW2 as well as CbiA from *B. miyamotoi* HT31, and the cloning of the Vsp1, Vlp15/16, and Vlp18 encoding genes of *B. miyamotoi* HT31 has been previously described^27,43,48,50,51^. To generate recombinant proteins carrying the identical hexahistidine tag at the N-terminus, the Vsp1, Vlp15/16, and Vlp18, respectively, encoding genes without the N-terminal signal sequence were re-cloned into pQE-30 Xa (Supplementary Table 1). For control purposes, a variant of the BBK32 protein encompassing amino acids 205 to 354 required for binding of complement C1r to maintain its inhibitory activity on the CP^87^ was also constructed. First, a DNA fragment encoding amino acid residues 21 to 354 of BBK32 of *B. burgdorferi* B31 was PCR-amplified from the pMalc-BBK32 vector using oligonucleotides BBK32 Bam_FP and BBK32 Hind_RP (Supplementary Table 1). After digestion, the DNA fragment was sub-cloned into pQE-30 Xa. To generate a truncated variant of BBK32, the generating vector pQE-BBK32 was used as template for amplifying a DNA fragment encoding residues 205 to 354 using oligonucleotides BBK32-205 BamHI and BBK32 Hind_RP. DNA fragments digested were then sub-cloned into pQE-30 Xa. Plasmids from selected clones were then purified and sequenced to ensure that the genes are cloned in frame and no random mutations have been introduced. The production of recombinant proteins in *E. coli* BL21(DE3) and their purification by affinity chromatography have previously been described^46^.

### SDS-PAGE, Western blotting, and silver staining

For separation of purified proteins, 10% Tris/Tricine SDS gels have been used^46^. Visualization of recombinant proteins by Western blotting applying anti-His antibodies and silver staining (Supplementary Fig. 4) were performed as described previously^46^.

### Enzyme-linked immunosorbent assay (ELISA)

To assess binding of plasminogen or FH, 96-well microtiter plates (MaxiSorp, Nunc) were coated with recombinant proteins or BSA (5 µg/ml) in 100 µl PBS at 4 °C overnight with gentle agitation as described recently^46,59^. Briefly, following blocking 100 µl plasminogen (10 µg/ml) or complement components FH, C3b, C5, and FB (5 µg/ml each) were added and the plates were incubated at room temperature for 1 h. The wells were subsequently washed and incubated for 1 h at room temperature with a polyclonal antiserum (1:1000) raised against human plasminogen or FH, C3b, C5, and FB respectively. After incubation with an HRP-conjugated anti-goat antiserum (1:2000), the plates were washed and developed with *o*-phenylenediamine (Sigma-Aldrich, Steinheim, Germany) and the absorbance was read at 490 nm (PowerWave HT, Bio-Tek Instruments, Winooski, VT, USA).

To determine dose-dependent binding of plasminogen and to calculate the dissociation constants, Vlp15/16, Vsp1, and BBA70 coated to the wells were incubated with increasing amounts of plasminogen (0 to 20 µM). In the presence of increasing concentrations of the lysine analog tranexamic acid (0 to 50 mM) or NaBr (0 to 1000 mM), plasminogen was added to the wells coated with either Vlp15/16, Vsp1 or BBA70 to determine the role of lysine residues and the effect of ionic strength on plasminogen binding as described previously^46^.

### Plasmin(ogen) activation assay

To analyse conversion of protein-bound plasminogen to active plasmin, cleavage of the chromogenic substrate D-Val-Leu-Lys-*p*-nitroanilide dihydrochloride was assayed as described previously^46,51^. In brief, microtiter plates coated with 100 µl of His_6_-tagged proteins or BSA (5 µg/ml each) in PBS were incubated with 10 µg/ml of glu-plasminogen for 1 h at room temperature. Following three wash steps, wells were incubated with a reaction mixture containing 50 mM Tris/HCl, pH 7.5, 300 mM NaCl, 0.003% Triton X-100, and 0.3 mg/ml S-2251. Finally, 4 µl of 2.5 µg/ml urokinase plasminogen activator (uPA) were added to each well to activate protein-bound plasminogen to plasmin. Plates were then sealed and placed in an ELISA reader and incubated at 37 °C for 24 h. The absorbance was measured every 30 min at 405 nm. Reaction mixtures containing 50 mM tranexamic acid or in which plasminogen or uPA were omitted served as controls.

### Complement inactivation assay

The inhibitory capacity of borrelial proteins on the CP, LP or the AP was analysed by a microtiter-based approach as described previously^59^. Briefly, microtiter plates were coated with either human IgM (30 ng/ml) (Merck, Darmstadt, Germany) for the CP, mannan (1 µg/ml) (Merck, Darmstadt, Germany) for the LP or LPS (100 ng/ml) (Hycult Biotech, Beutelsbach, Germany), for the AP at 4 °C overnight. Following blocking, NHS (1% for the CP, 2% for the LP, and 15% for the AP) pre-incubated with His_6_-tagged proteins (10 µg each or increasing concentrations thereof) were added to initiate complement activation. Formation of the MAC was detected by using an anti-C5b-9 antibody (1:500) (Quidel, Athens, USA) and antigen–antibody complexes were visualized by applying HRP-conjugated anti-mouse immunoglobulins (1:1000). The reactions were developed by adding *o-*phenylenediamine (Merck, Darmstadt, Germany) and measuring the absorbance at 490 nm.

### Serum bactericidal assay

Protection from complement-mediated lysis mediated by recombinant proteins was assessed by pre-incubation of 50 µl human serum with 4 µM of either Vlp15/16, Vlp18, CspA or BSA for 15 min at 37 °C with gentle agitation. The pre-incubated serum samples were then adjusted to 100 µl with BSK-H medium. As additional reaction mixtures, native NHS (not pre-treated), heat-inactivated NHS and a Tris/HCl-buffer control were also included. In parallel, 1 × 10^8^ spirochetes of serum sensitive *B. garinii* strain G1 were sedimented by centrifugation and resuspended in the pre-treated serum samples as well as the controls. All reaction mixtures were then incubated for 6 h at 37 °C with gentle agitation. The percentage of motile and viable cells was determined by dark field microscopy at every hour as described previously^59^. Spirochetes in nine microscopy fields were counted by using Glasstic slides 10 (KOVA International Inc., CA, USA). Each test was performed three times and ± SEM was determined by using GraphPad Prism version 7.

### Sequence analysis

Vectors constructed were purified and sequenced by using a commercial sequencing service (Eurofins Genomics, Germany GmbH, Ebersberg, Germany). For bioinformatic analyses, the CLC sequence Viewer 8.0 (QIAGEN Aarhus A/S, Denmark) was utilized.

### Statistical analysis

The data collected represent means from at least three independent experiments, and error bars indicate SD. For statistical analyses, one-way ANOVA with Bonferroni’s multiple comparison post-hoc test (95% confidence interval) were conducted by applying GraphPad Prism version 7.

### Ethics statement

Collection of blood samples and consent documents were approved by the ethics committee at the University Hospital of Frankfurt (control number 160/10 and 222/14), Goethe University of Frankfurt am Main. All healthy blood donors provided written informed consent in accordance with the Declaration of Helsinki.

## Supplementary Information

Supplementary Information.

## Data Availability

The datasets generated during and/or analysed during the current study are available from the corresponding author on reasonable request.
