# Supplementary material for: Interaction between Borrelia miyamotoi variable major proteins Vlp15/16 and Vlp18 with plasminogen and complement
Source: Sci Rep. 2021 Mar 2;11:4964. doi: 10.1038/s41598-021-84533-x (PMC7925540; doi:10.1038/s41598-021-84533-x)
Supplement: Supplementary file 1 — Supplementary Information. [file 41598_2021_84533_MOESM1_ESM.pdf]

Supplementary Information

Interaction between *Borrelia miyamotoi* variable major proteins Vlp15/16 and Vlp18 with plasminogen and complement

Frederik L. Schmidt[a], Valerie Sürth[a], Tim K. Berg[a], Yi-Pin Lin[b,c], Joppe W. Hovius[d], and Peter Kraiczy[a]

[a]Institute of Medical Microbiology and Infection Control, University Hospital of Frankfurt, Goethe University Frankfurt, Germany

[b]Division of Infectious Diseases, Wadsworth Center, New York State Department of Health, Albany, New York, USA

[c]Department of Biomedical Science, State University of New York at Albany, New York, USA

[d]Center for Experimental and Molecular Medicine, Academic Medical Center, Amsterdam Infection and Immunity Institute, Amsterdam, The Netherlands

Corresponding author: Dr. Peter Kraiczy, Institute of Medical Microbiology and Infection Control, University Hospital of Frankfurt, Paul-Ehrlich-Str. 40, D-60596 Frankfurt, Germany, Phone: +49-69-6301-7165, E-mail: Kraiczy@em.uni-frankfurt.de

Supplementary Figure 1

Vlp15/16 protein of *B. miyamotoi* LB-2001 (WP_025444482.1)

HHHHHHGSGSGSGIEGRPYNGTGSNNGGGEDPQKVFLTSIANLGKGFLDVFVTFGDMVTGAFGIKAET
KKSDVGQYFTSIAETMESVKKKLQDEVAANGNYEKVKTVVEQFITGTLDKIAAGAKEAAKGATTDAAIGN
ATSTGHGATPANKESVVSLVKGMKTIVDVVLKDKGDASATKTGDDKKDIGKLFTDDAGKADAKEENIAK
AAASIGAVTGADVLQAIVQSNENPTADSTNGIEKAKDAAEIAIAPAVNNKKEIKEVSAKKDAIIAAGIALRA
MAKGGKFAANNDAKDADAVNGAAASAVGKTLSTLIIAVRNTVDSGLKKINEVLATVKQEDKSVEATKTA
EVTTSAQK

The deduced protein sequence of the pQE-30 Xa vector including the hexahistidine tag, the factor Xa splicing site, and the multiple cloning site is highlighted in light blue and the lysine residues in Vlp15/16 are indicated in green. The Vlp15/16 protein consists of 42 lysine residues. The *vlp15/16* gene lacks the N-terminal nucleotides encoding for 20 amino acid residues (MSKRKTLSAIIMTLFLIIGC).

Supplementary Figure 2

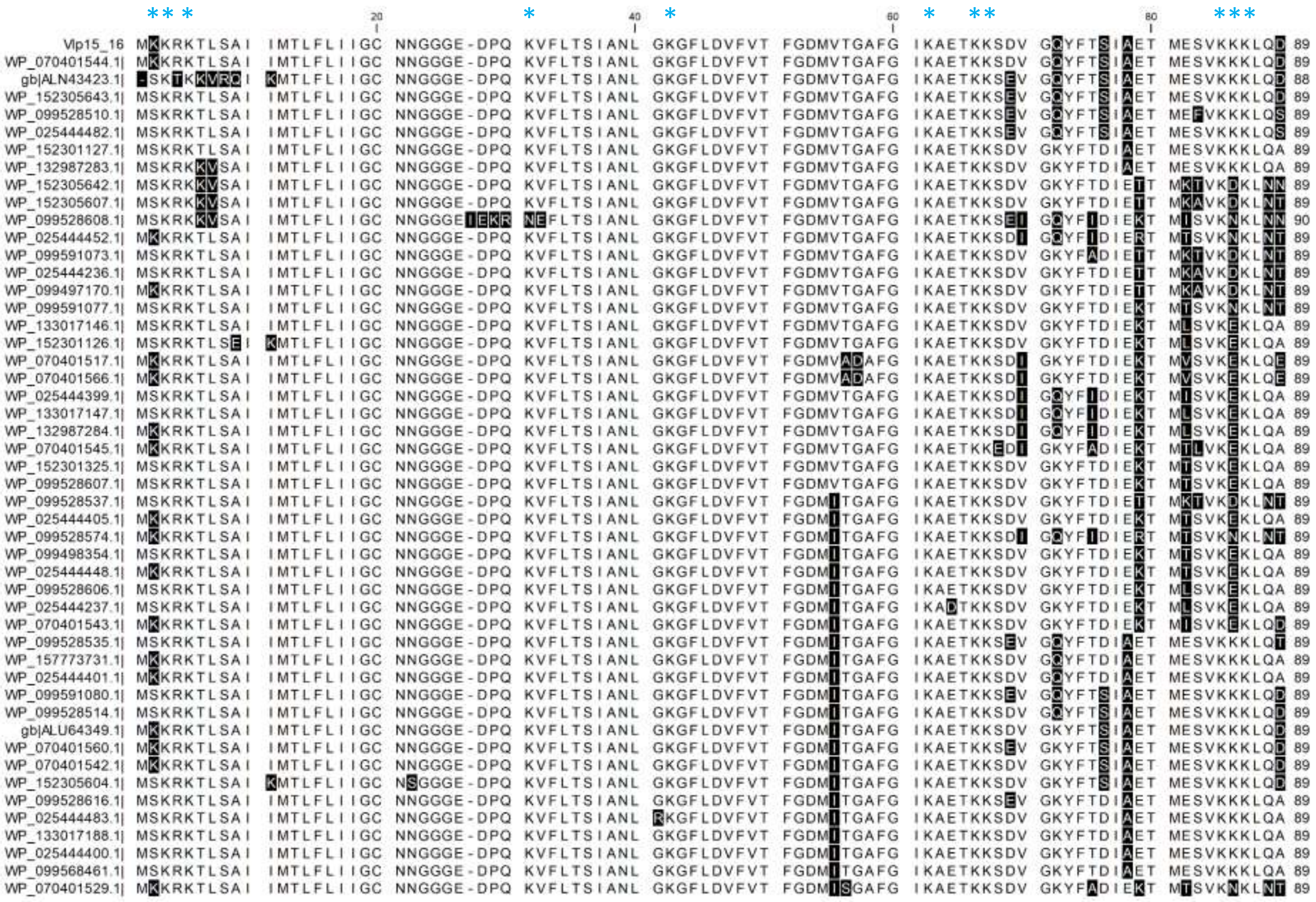

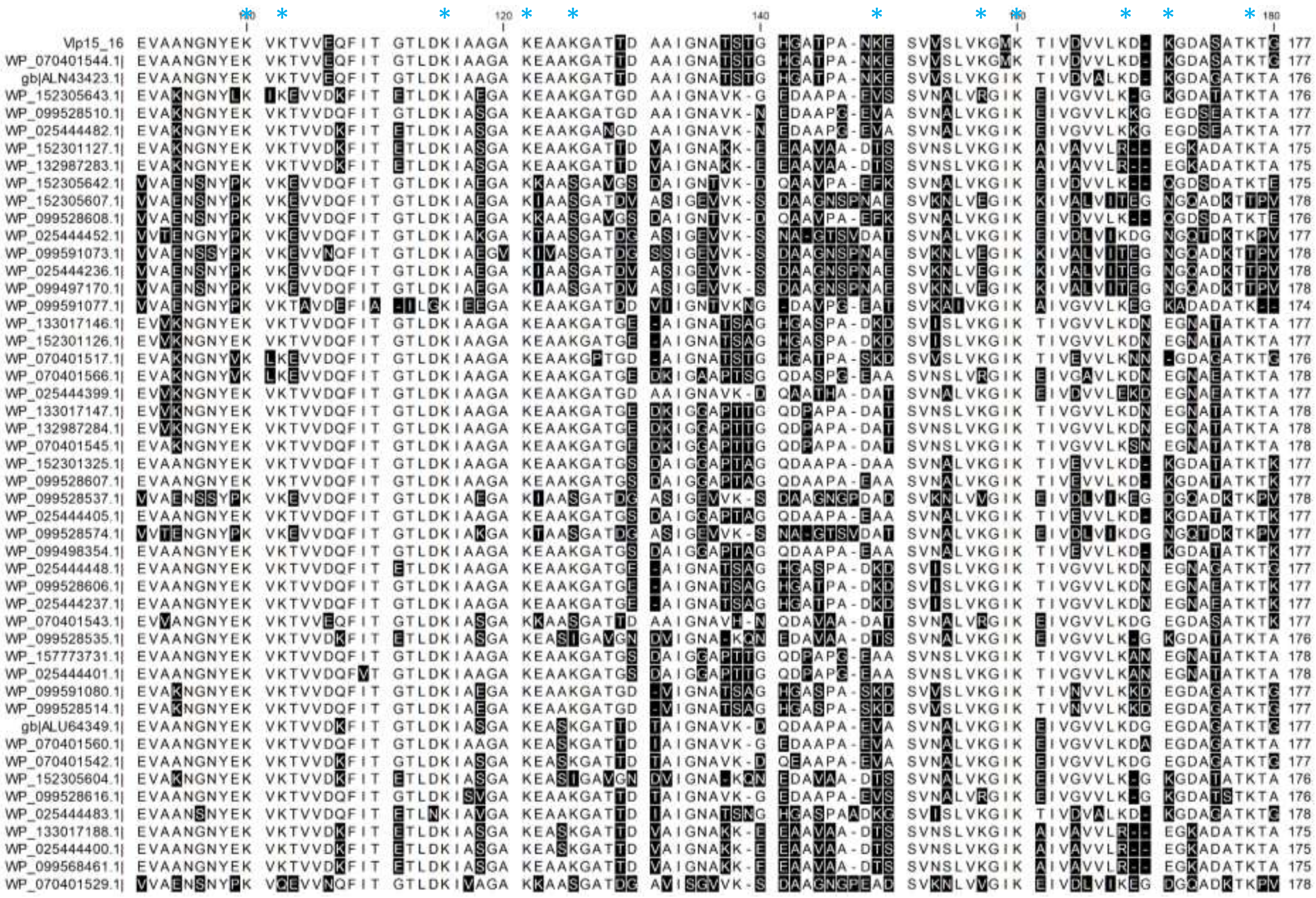

110
120
140
160
180
Vlp15_16 177
WP_070401544.1| 177
gb|ALN43423.1| 176
WP_152305643.1| 176
WP_099528510.1| 177
WP_025444482.1| 177
WP_152301127.1| 175
WP_132987283.1| 175
WP_152305642.1| 175
WP_152305607.1| 178
WP_099528608.1| 176
WP_025444452.1| 177
WP_099591073.1| 178
WP_025444236.1| 178
WP_099497170.1| 178
WP_099591077.1| 174
WP_133017146.1| 177
WP_152301126.1| 177
WP_070401517.1| 176
WP_070401566.1| 178
WP_025444399.1| 177
WP_133017147.1| 178
WP_132987284.1| 178
WP_070401545.1| 178
WP_152301325.1| 177
WP_099528607.1| 177
WP_099528537.1| 178
WP_025444405.1| 177
WP_099528574.1| 177
WP_099498354.1| 177
WP_025444448.1| 177
WP_099528606.1| 177
WP_025444237.1| 177
WP_070401543.1| 177
WP_099528535.1| 176
WP_157773731.1| 178
WP_025444401.1| 178
WP_099591080.1| 177
WP_099528514.1| 177
gb|ALU64349.1| 177
WP_070401560.1| 177
WP_070401542.1| 177
WP_152305604.1| 176
WP_099528616.1| 176
WP_025444483.1| 178
WP_133017188.1| 175
WP_025444400.1| 175
WP_099568461.1| 175
WP_070401529.1| 178

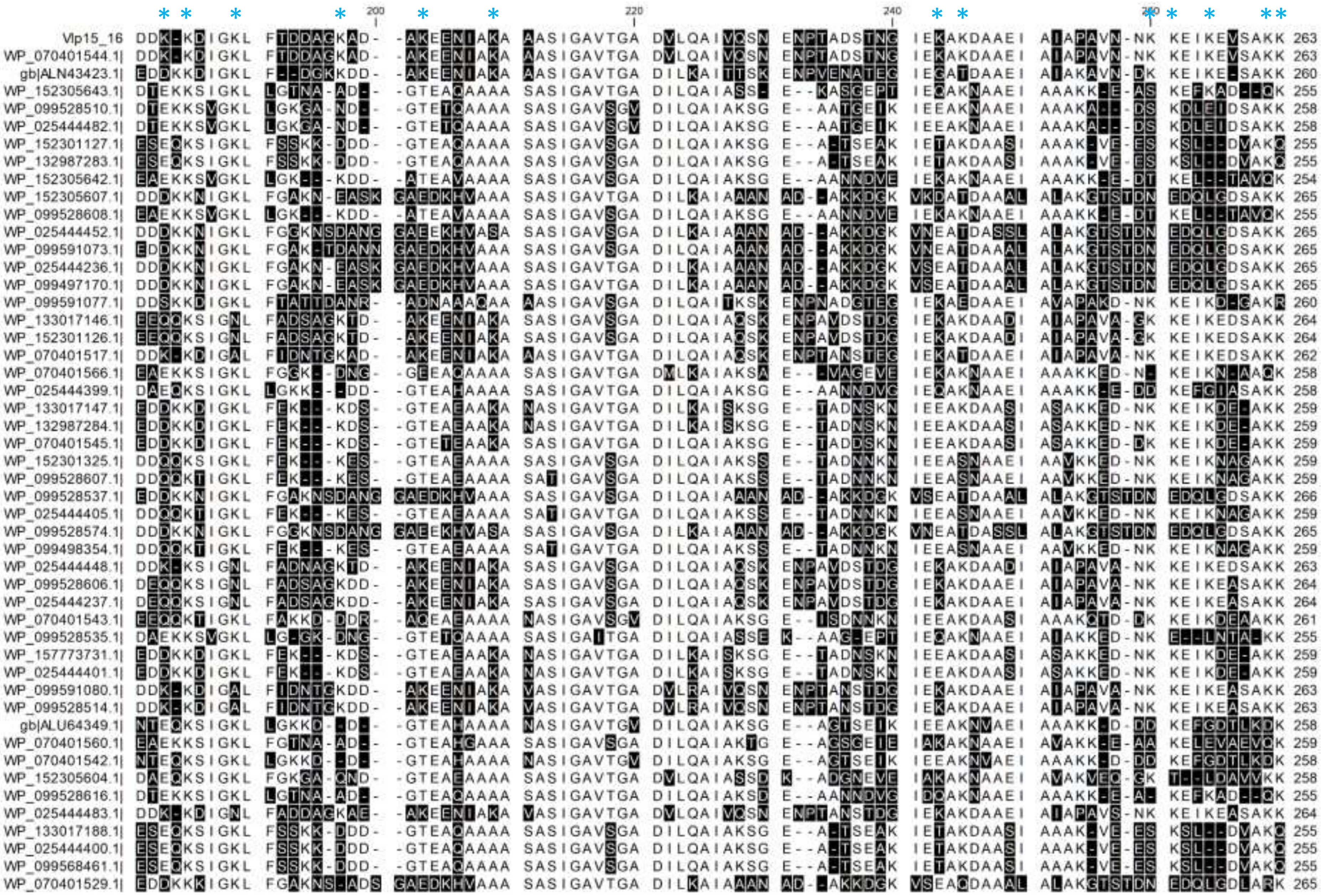
200
220
240
260
Vlp15_16 263
WP_070401544.1| 263
gb|ALN43423.1| 260
WP_152305643.1| 255
WP_099528510.1| 258
WP_025444482.1| 258
WP_152301127.1| 255
WP_132987283.1| 255
WP_152305642.1| 254
WP_152305607.1| 265
WP_099528608.1| 255
WP_025444452.1| 265
WP_099591073.1| 265
WP_025444236.1| 265
WP_099497170.1| 265
WP_099591077.1| 260
WP_133017146.1| 264
WP_152301126.1| 264
WP_070401517.1| 262
WP_070401566.1| 258
WP_025444399.1| 258
WP_133017147.1| 259
WP_132987284.1| 259
WP_070401545.1| 259
WP_152301325.1| 259
WP_099528607.1| 259
WP_099528537.1| 266
WP_025444405.1| 259
WP_099528574.1| 265
WP_099498354.1| 259
WP_025444448.1| 263
WP_099528606.1| 264
WP_025444237.1| 264
WP_070401543.1| 261
WP_099528535.1| 255
WP_157773731.1| 259
WP_025444401.1| 259
WP_099591080.1| 263
WP_099528514.1| 263
gb|ALU64349.1| 258
WP_070401560.1| 259
WP_070401542.1| 258
WP_152305604.1| 258
WP_099528616.1| 255
WP_025444483.1| 264
WP_133017188.1| 255
WP_025444400.1| 255
WP_099568461.1| 255
WP_070401529.1| 265

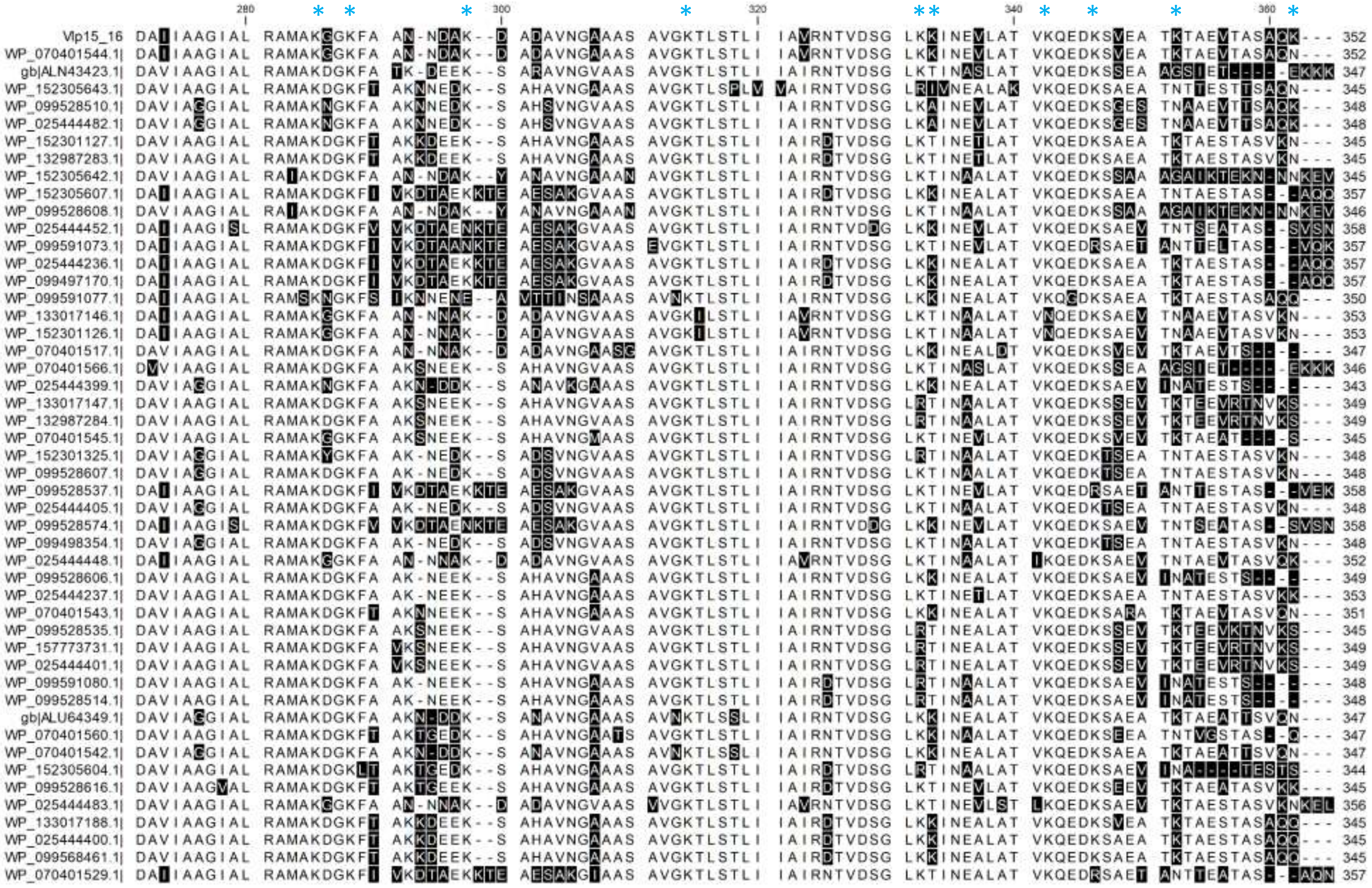

Deduced amino acid alignment of diverse Vlp orthologs (δ-subfamily) of *B. miyamotoi* (taxid:47466). Only full sequences have been used to created the alignment by employing the Constraint-based Multiple Alignment Tool of BLASTp (COBALT). Residues differing from the Vlp15/16 sequence of strain LB-2001 are boxed and the lysine residues are marked by an asterisk.

Supplementary Figure 3

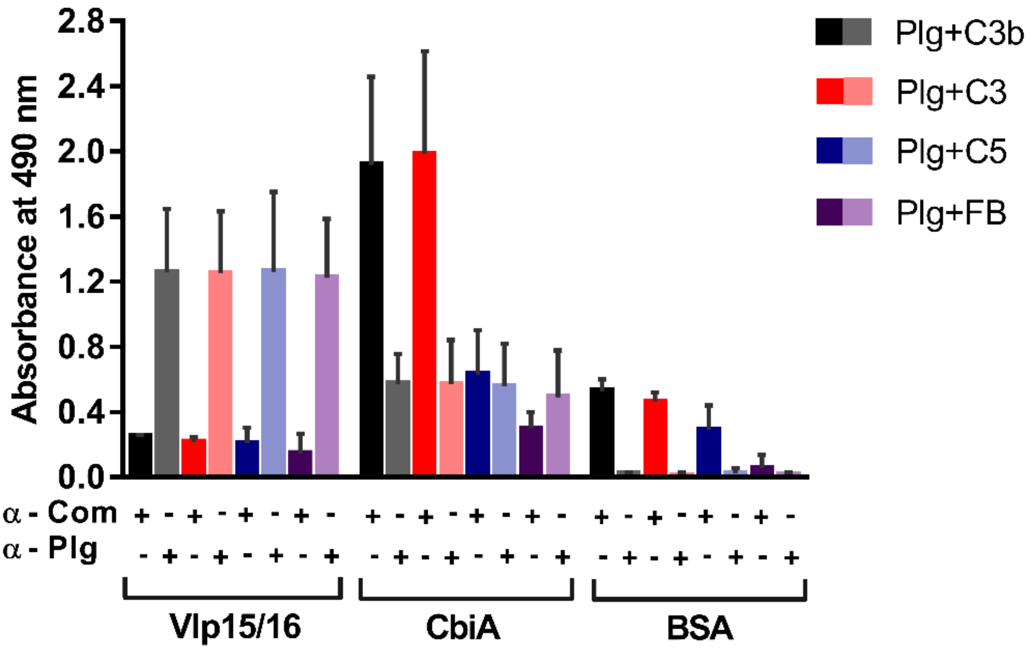

Binding of complement components to plasminogen bound to Vlp15/16. To assess binding of complement components C3b, C3, C5 and Factor B (FB) to plasminogen bound to Vlp15/16, 96-well microtiter plates (MaxiSorp, Nunc) were coated with 0.1 µM of Vlp15/16, CbiA (additional control), and BSA (negative control), respectively, in 100 µl PBS at 4 °C overnight. Following blocking with 100 µl PBS containing 0.2% (w/v) gelatin. For each reaction mixtures, two duplicates were prepared to detect binding of plasminogen (Plg) or binding of complement components (Com) to plasminogen bound to Vlp15/16, CbiA, and BSA, respectively. In addition, four duplicates of each protein were coated to serve as primary antibody controls and where the ligands were omitted. The respective wells were subsequently washed and incubated with 0.1 µM plasminogen in 100 µl PBS for 1 h at room temperature. Afterwards, complement components C3b, C3, C5, and FB were added to the respective wells at a concentration of 0.1 µM in 100 µl PBS and incubated for 1 h at RT. The wells were then washed twice and binding of the ligands was detected by using a polyclonal antiserum (1:1,000) raised against human plasminogen or C3, C5, and FB, respectively. After incubation with a HRP-conjugated anti-goat antiserum (1:2,000), the plates were washed and developed with *o*-phenylenediamine (Sigma-Aldrich, Steinheim, Germany) for 8 min and the absorbance was measured at 490 nm (PowerWave HT, Bio-Tek Instruments, Winooski, VT, USA). Finally, the values of the antibody controls were subtracted from the values obtained from the reaction mixtures containing plasminogen and complement components. Data represent means and standard deviation of at least two independent experiments, each conducted in duplicate. For visualization of the data, GraphPad Prism 7 was conducted.

Supplementary Figure 4

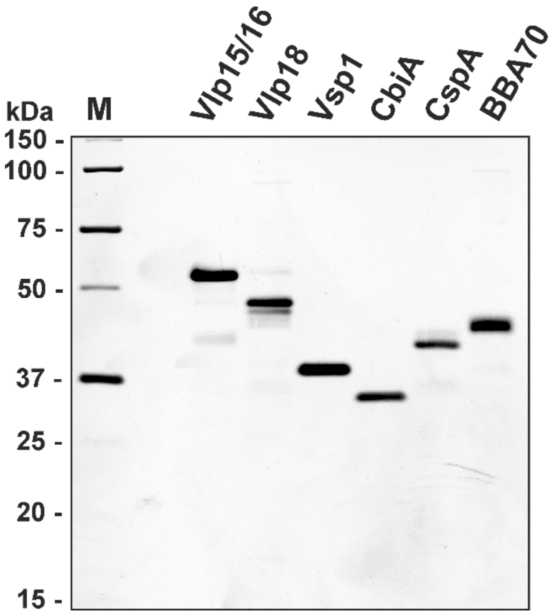

Characterization of purified His_6-tagged proteins by silver staining. Purified Vlp15/16, Vlp18, Vsp1, CbiA, CspA, and BBA70 (1 µg each) were separated by a 10% Tris/Tricine-SDS-gel and stained with silver nitrate. The molecular weight markers (M) Precision Plus Protein Standards, Bio-Rad) are indicated on the left. The gel was scanned using a GS-900 Imaging Densitometer (Bio-Rad) and for image processing the Image Lab software, Version 6.1.0 (Bio-Rad) was applied. The figure shows the original version of the scanned gel. The general settings were as follows: Application: silver gel; Filter setting: Silver stain; Resolution: 63.5 * 63.5 microns.

Supplementary table 1. Oligonucleotides used in this study

Oligonucleotide	Sequence (5'-3')[a]	Use in this work
Vlp15/16_FP	GATATACATATGGCTGGATCCAATAATGGAGGAGGGGAAG	Recloning of *vlp15/16* into pQE-30Xa vector
Vlp15/16_RP	CTGCAGGTCGACTTATTACTTCTGTGCACTAGTTGTTAC	Recloning of *vlp15/16* into pQE-30Xa vector
Vlp18_FP	CAACAAACAGAGGATCCGTATCAGGAGGAGATAAACAAGGGGTTG	Recloning of *vlp18* into pQE-30Xa vector
Vlp18_RP	CTGCAGGTCGACTTATTACTCTGCTGTTTTTGAGTTTCTTG	Recloning of *vlp18* into pQE-30Xa vector
BmVsp1_Bam	CATATGGCTAGCTGTGGATCCGGGGGACCGGCACC	Recloning of *vsp1* into pQE-30Xa vector
BmVsp1_Sal	GTGGTGGTGGTGGTCGACCTATTATGAAGATTGACCAGCAG	Recloning of *vsp1* into pQE-30Xa vector

[a] Sequences of specific restriction endonuclease recognition sites are underlined
